# Light Directs Zebrafish *period2* Expression via Conserved D and E Boxes

**DOI:** 10.1371/journal.pbio.1000223

**Published:** 2009-10-27

**Authors:** Gad Vatine, Daniela Vallone, Lior Appelbaum, Philipp Mracek, Zohar Ben-Moshe, Kajori Lahiri, Yoav Gothilf, Nicholas S. Foulkes

**Affiliations:** 1 Department of Neurobiology, George S. Wise Faculty of Life Sciences, Tel Aviv University, Tel Aviv, Israel; 2 Institute of Toxicology and Genetics, Karlsruhe Institute of Technology, Eggenstein-Leopoldshafen, Germany; Charité - Universitätsmedizin Berlin, Germany

## Abstract

For most species, light represents the principal environmental signal for entraining the endogenous circadian clock. The zebrafish is a fascinating vertebrate model for studying this process since unlike mammals, direct exposure of most of its tissues to light leads to local clock entrainment. Importantly, light induces the expression of a set of genes including certain clock genes in most zebrafish cell types in vivo and in vitro. However, the mechanism linking light to gene expression remains poorly understood. To elucidate this key mechanism, here we focus on how light regulates transcription of the zebrafish *period2* (*per2*) gene. Using transgenic fish and stably transfected cell line–based assays, we define a Light Responsive Module (LRM) within the *per2* promoter. The LRM lies proximal to the transcription start site and is both necessary and sufficient for light-driven gene expression and also for a light-dependent circadian clock regulation. Curiously, the LRM sequence is strongly conserved in other vertebrate *per2* genes, even in species lacking directly light-sensitive peripheral clocks. Furthermore, we reveal that the human LRM can substitute for the zebrafish LRM to confer light-regulated transcription in zebrafish cells. The LRM contains E- and D-box elements that are critical for its function. While the E-box directs circadian clock regulation by mediating BMAL/CLOCK activity, the D-box confers light-driven expression. The zebrafish homolog of the thyrotroph embryonic factor binds efficiently to the LRM D-box and transactivates expression. We demonstrate that *tef* mRNA levels are light inducible and that knock-down of *tef* expression attenuates light-driven transcription from the *per2* promoter in vivo. Together, our results support a model where a light-dependent crosstalk between E- and D-box binding factors is a central determinant of *per2* expression. These findings extend the general understanding of the mechanism whereby the clock is entrained by light and how the regulation of clock gene expression by light has evolved in vertebrates.

## Abstract 2

A highly conserved promoter module in a vertebrate clock gene confers light-regulated gene expression.

## Author Summary

Light is the principal signal used by animals to synchronize their circadian clocks with the day/night environment. Central to this vital property is the ability of light to trigger changes in gene expression. However, we still lack a complete understanding of how this occurs. The zebrafish is particularly interesting in this regard since direct light exposure induces the expression of clock genes in most of its tissues and in turn adjusts the phase of the intrinsic clocks. Here, by studying the promoter of one key light-regulated zebrafish clock gene, *per2*, we have identified a Light Responsive Module (LRM) that is necessary and sufficient for light controlled expression. Interestingly, the LRM is also highly conserved in the *per2* genes of other vertebrates that lack widespread light-sensing tissues. In addition, the human LRM can substitute for its zebrafish counterpart to confer direct light regulation of gene expression in zebrafish cells. The LRM contains E- and D-box enhancers critical for its function. While the E-box is a target of clock regulation, the D-box directs light driven expression. We show that the expression of the D-box binding transcription factor, *tef*, is itself induced by light and is essential for normal light-induced *per2* expression. These results advance our understanding of the mechanisms underlying entrainment by light and how light-regulated clock gene expression has evolved in vertebrates.

## Introduction

Essentially all organisms demonstrate daily rhythms in biochemistry, physiology, and behaviour that are controlled by the circadian clock [1]. Underlying this oscillator is a molecular mechanism consisting of interacting positive and negative feedback loops that function in a cell-autonomous manner [2]. As the free running period of the clock is not precisely 24 h, it must be reset each day by external signals in order to remain synchronized with the photoperiod of the environment [3]. The principal external cue that entrains circadian rhythmicity is light [4]. In mammals, photic signals from the eyes are communicated via the retino-hypothalamic tract (RHT) to the master circadian pacemaker in the suprachiasmatic nucleus (SCN) and thereby they synchronize rhythmic neuronal activity [1]. The SCN in turn coordinates an array of peripheral oscillators, hence driving a diversity of rhythmic processes such as locomotor activity, metabolism, and hormonal secretion. One extensively studied SCN-driven process is the rhythmic production of melatonin in the pineal gland. Via a multisynaptic pathway, SCN neurons stimulate the production of melatonin at night by activating cAMP production [5].

In non-mammalian vertebrates, the circadian timing system appears to perceive light through a more distributed photoreceptive system. Thus, outside of the retina, dedicated photoreceptors are also encountered in the pineal gland, the parietal eye, as well as deep brain structures [6]. The pineal gland of non-mammalian vertebrates contains a photoreceptive circadian oscillator that directly drives rhythmic melatonin production. Consequently, cultured pineal glands are capable of autonomously generating a melatonin rhythm that can be entrained by light [6]–[8]. In some species the pineal gland is considered to act as a master circadian pacemaker [6]. Interestingly, in zebrafish, circadian clocks within many tissues and cell lines have also been shown to be directly light entrainable [9]–[11]. The mechanism whereby light directly synchronizes the rhythms of these peripheral clocks is still a mystery.

As demonstrated in several species, *period* (*per*) genes, negative components of the circadian oscillator, are involved in its entrainment by light. *Per1* and *per2* mRNA levels in the rodent SCN are increased by light exposure during the subjective night but not during the subjective day [12]. Moreover, mice carrying mutated *per1* or *per2* genes do not exhibit the normal advanced and delayed phase shift responses to light pulses [13]. The *per2* transcript has also been shown to be light inducible in the chicken pineal gland and SCN [14]. Furthermore, in *Xenopus*, expression of *per2* in the retina and retinal pigment epithelium is light-dependent and is therefore suggested to play a role in circadian entrainment [15].

In zebrafish, the expression pattern of *per* genes has been studied both in vitro and in vivo. In zebrafish cell lines (PAC-2, Z3), *per1* (also termed *per4*), *per2*, and *per3* transcripts have been shown to exhibit a robust oscillation under light/dark (LD) cycles [9],[16],[17]. Expression of *per1* and *per3* seems to be driven by a circadian oscillator since their rhythmic expression persists following transfer to constant darkness (DD) and their daily increase in transcript levels anticipates the light phase [9],[16],[17]. In contrast, *per2* mRNA levels are thought to increase only when cells are exposed to light and its rhythmic expression dampens immediately following transfer to DD. Consistently, in zebrafish embryos, *per2* mRNA expression is induced in response to light exposure throughout the body, cranial areas and particularly in the pineal gland [18]. Pineal *per2* mRNA levels increase rapidly following “lights on”, reaching a peak after 3 h while they remain undetectable under DD. Moreover, knock-down analysis has demonstrated that *per2* expression is required for the light-induced developmental maturation of the pineal clock [18]. These studies indicate that *per2* is involved in the light-input pathway of the circadian clock in zebrafish. Furthermore, the synchronization and onset of circadian rhythmicity in the pineal gland depends on light induction of *per2* expression. The mechanism by which light regulates *per2* expression is unknown.

With the goal of improving our understanding of the mechanisms underlying light entrainment in the vertebrate circadian clock, we investigated the regulation of zebrafish *per2* both in vivo and in vitro. The *per2* promoter regulatory region was identified and two distinct transcription factor control mechanisms were shown to contribute to light-driven *per2* expression. Our results point to a crosstalk between circadian clock and light-driven regulation as being a key feature of the *per2* gene regulation.

## Results

### Identification of the *per2* Promoter

**Figure 1:**
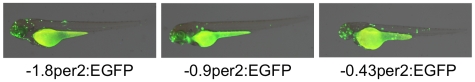
Transient expression of EGFP under the control of the *per2* promoter. Representative photographs of 3 day(s) post-fertilization (dpf) microinjected larvae. Left panel: −*1.8per2:EGFP* construct was microinjected into zebrafish embryos. Ninety-four percent of injected embryos were EGFP-positive, exhibiting robust EGFP expression scattered throughout all tissues. Injection of −*0.9per2:EGFP* (middle panel) and −*0.43per2:EGFP* (right panel) embryos resulted in similar percentages of EGFP-positives, expression patterns, and intensities.

The *per2* gene plays a key role in mediating the effects of light on the circadian clock in zebrafish [18]. An important step towards exploring the mechanisms whereby light regulates expression of this gene is to isolate and characterize its promoter. A genomic fragment incorporating the putative promoter (1.8 kb 5′ flanking genomic DNA) and the first exon (164 bp) of *per2* was subcloned upstream of an EGFP reporter gene creating the −*1.8per2:EGFP* construct. Microinjection of −*1.8per2:EGFP* resulted in 94% EGFP-positive embryos, each exhibiting robust transient EGFP expression scattered throughout the body without any apparent tissue specificity as seen under a fluorescent dissecting microscope (Figure 1). This EGFP expression pattern is consistent with the documented widespread expression of zebrafish *per2* mRNA in zebrafish embryos and larvae [18]. In order to identify the minimal promoter required for expression, two additional shorter constructs were tested by microinjection. Embryos injected with −*0.9per2:EGFP* and −*0.43per2:EGFP* resulted in 93% and 94% EGFP-positive embryos, respectively. Expression patterns and intensities were similar to those observed with the −*1.8per2:EGFP*-injected embryos (Figure 1). These results indicate that the 430 bp 5′-flanking region minimal promoter is sufficient to drive expression.

### The *per2* Promoter Drives Ubiquitous Expression that is Enhanced in the Pineal Gland

**Figure 2:**
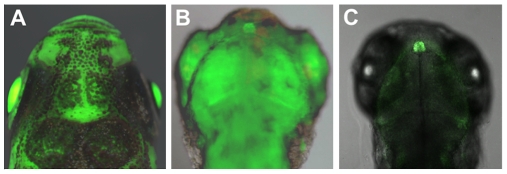
EGFP expression in *Tg(*–*0.43per2:EGFP)tlv1*. The *per2* minimal promoter drives an EGFP expression throughout all tissues that is augmented in the pineal gland. Transgenic *Tg(*–*0.43per2:EGFP)tlv1* adult (A) and 3 dpf larva (B) under a stereo dissecting microscope. 2 dpf embryo (C) under a confocal microscope. See also Video S1 and Figure S1.

Using the *Tol2* transposon system [19], a transgenic line, *Tg(*−*0.43per2:EGFP)tlv1* containing the 430 bp promoter fragment fused to EGFP, was generated. This line exhibits ubiquitous EGFP expression with enhanced expression in the pineal gland in embryos, larvae, and adults (Figure 2 and Video S1). This expression pattern is in accordance with previous whole mount in situ hybridization (ISH) analyses for *per2* [18]. *Tg(*−*0.43per2:EGFP)tlv1* were crossed with *Tg(aanat2:mRFP)y164* (kindly provided by Reiko Toyama, NIH), which exhibits red fluorescence exclusively in the pineal gland, to create a double transgenic line. Confocal in vivo analysis reveals co-localized EGFP and mRFP expression in the pineal gland (Figure S1). These results indicate that *per2* is expressed in virtually all cells that contain an autonomous peripheral circadian oscillator and exhibits enhanced expression in the melatonin-producing, master clock-containing, photoreceptor cells of the pineal gland.

### The *per2* Promoter is Light Responsive In Vivo

**Figure 3:**
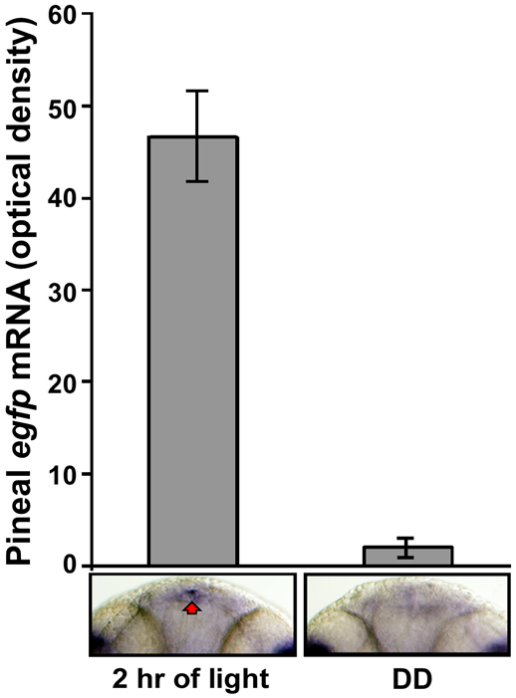
The *per2* minimal promoter is light-induced in vivo: quantification of pineal EGFP mRNA levels. *Tg(*–*0.43per2:EGFP)tlv1* embryos were entrained by exposure to two LD cycles. At the beginning of the third day of development, embryos were either exposed to light or kept in darkness. After 2 h (ZT 2 and CT 2) embryos were fixed and subjected to whole mount ISH for *egfp* mRNA. Signal intensities, determined using ImageJ software, revealed a 22-fold increase in pineal *egfp* mRNA levels after a 2 h light pulse (*n* = 16, *p*<0.01 by ANOVA) relative to pineal *egfp* expression in the DD controls. Error bars represent SE. Dorsal views of representative whole mounts are shown below.

To determine whether the −*0.43 kb per2* promoter is light responsive in vivo, *Tg(*−*0.43per2:EGFP)tlv1* embryos were kept under LD cycles during the first 2 d of development. On the third day of development, entrained embryos were either exposed to 2 h of light at ZT 0 or maintained in DD. Differences in EGFP fluorescence were not observed, most likely due to the stability of the EGFP protein. However, analysis of *egfp* mRNA using whole mount ISH revealed increased expression of the transgene after light exposure, notably in the pineal gland. Quantification of the pineal signal revealed a 22-fold increase in *egfp* mRNA levels after a 2 h light pulse with respect to a DD control (*n* = 16, *p*<0.05 by one-way ANOVA) (Figure 3). These results are in agreement with those shown previously by whole mount ISH for the endogenous *per2* mRNA [18] and indicate that the −*0.43per2* promoter directs light-induced expression in vivo.

### The *per2* Promoter Is Light-Responsive and Clock-Driven

**Figure 4:**
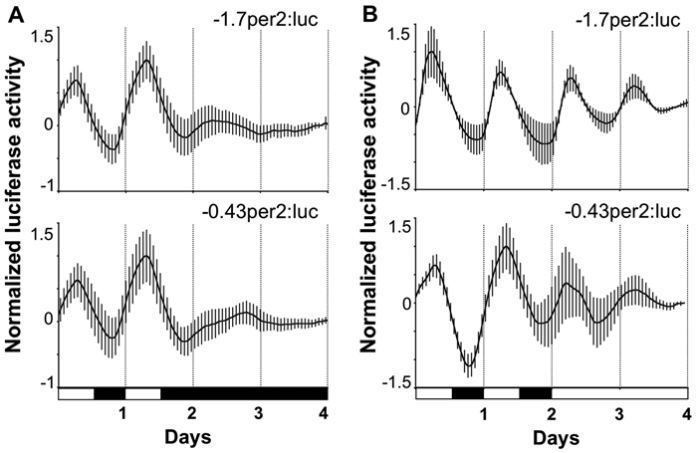
The *per2* minimal promoter is clock driven under LL conditions in vitro. Bioluminescence assay of cells stably transfected with −*1.7per2:luc* and −*0.43per2:luc*. Cells were maintained under LD and then transferred to DD (A) or LL (B). Relative bioluminescence is plotted on the *y*-axis and time (days) on the *x*-axis. For each point, error bars (grey) represent the SD. White/black bars show the light and dark periods, respectively. Both −1.7 and −0.43 kb promoters drive rhythmic luciferase expression under LD and LL but not under DD. These results indicate that, in addition to being light responsive, the *per2* promoter is clock driven under LL conditions.

Light-inducible *per2* expression and a directly light-entrainable clock have been encountered in several zebrafish cell lines [9],[17]. Stable transfection of these cells with luciferase reporter constructs followed by monitoring expression under various lighting conditions with a live cell bioluminescence assay represents a powerful approach to assess a promoter's light responsiveness [17]. Thus, we chose to stably transfect PAC-2 zebrafish cells with constructs that contain the *per2* promoter cloned upstream of the luciferase gene (−*1.7per2:Luc* and −*0.43per2:Luc*). Subsequently bioluminescence was monitored in living cells under LD conditions for 48 h followed by 48 h under DD, or constant light (LL). Under LD conditions, the 0.43 and 1.7 kb *per2* promoters drove rhythms of luciferase activity (τ = 24.1±0.3 h and τ = 23.9±0.8 h, respectively; Table 1). Expression of the reporter constructs increased during the beginning of the light phase (peaking at ZT 5.3±0.2 and 6.4±0.4, respectively) and then decreased during the dark phase (Figure 4). These results indicate that the minimal *per2* promoter is light-responsive in PAC-2 cells. Consistently, transfer to DD leads to a very rapid attenuation of rhythmic expression. Interestingly, under LD conditions the decrease in luciferase activity observed during the cycling anticipates the end of the light phase. In addition, a slight increase in luciferase activity is observed before the beginning of the light phase. Importantly, rhythmicity driven by the minimal promoter was maintained under LL (τ = 23.25±0.57 h) (Figure 4B), but not when cells were transferred to DD (Figure 4A). These results indicate that, in addition to being light driven, the *per2* promoter also shows regulation by the circadian clock.

**Table 1:** Tau and Peak values of constructs tested in PAC-2 cells under LD.

Construct	τ	Peak (CT)
−1.7per2:Luc	23.9±0.8	6.4±0.4
−0.43per2:Luc	24.1±0.3	5.3±0.2
Deletion 3, (Δ−276/−201)	24.3±0.9	5.9±0.6
Deletion 4, (Δ−233/−143)	30.3±9.4	13.9±9.5
Deletion 5, (Δ−163/−89)	23.4±1.9	21.5±1.9
Deletion 6, (Δ−102/−31)	24.7±0.7	5.9±0.7
Deletion 4.1, (Δ−231/−212)	24±1.2	5.3±0.7
Deletion 4.2, (Δ−216/−197)	24.2±0.8	5.3±0.7
Deletion 4.3, (Δ−199/−181)	23.9±0.5	5.3±0.4
Deletion 4.4, (Δ−184/−169)	26±11.5	9.4±6.3
Deletion 4.5, (Δ−170/−149)	26.7±11	9.4±6.3
Deletion 5.1, (Δ−155/−133)	16.3±1.7	1.8±1.7
Deletion 5.2, (Δ−134/−119)	24±1.2	5.4±0.4
Deletion 5.3, (Δ−121/−100)	23.9±1.3	5.4±0.7
Deletion 5.4, (Δ−225/−206)	24±1.2	5.3±0.6
Pluc MCS	36±10.2	8.8±9.2
3xLRM(fwd):Luc	23.9±0.3	5.3±0.2
3xLRM(rev):Luc	23.9±0.3	5.2±0.3
−0.43per2(hLRM):Luc	24.3±0.6	6.1±0.7
−0.43per2-ME:Luc	16.6±13.6	15.2±7.8
−0.43per2-MD:Luc	23.2±17.8	7.1±8.6
−0.43per2-ME-MD:Luc	21.5±12.5	13.7±6.9
−0.43per2-Mccaat:Luc	24±0.8	5.3±0.8
−0.43per2-Mcatgg:Luc	23.9±0.9	5.3±0.9
4xE-box	24.2±1.1	4.6±0.4
6xD-box	23.75±1.1	11.6±1
4xE/D-box	23.4±0.8	7±0.8

### Identification of a Light-Responsive Region within the *per2* Promoter

**Figure 5:**
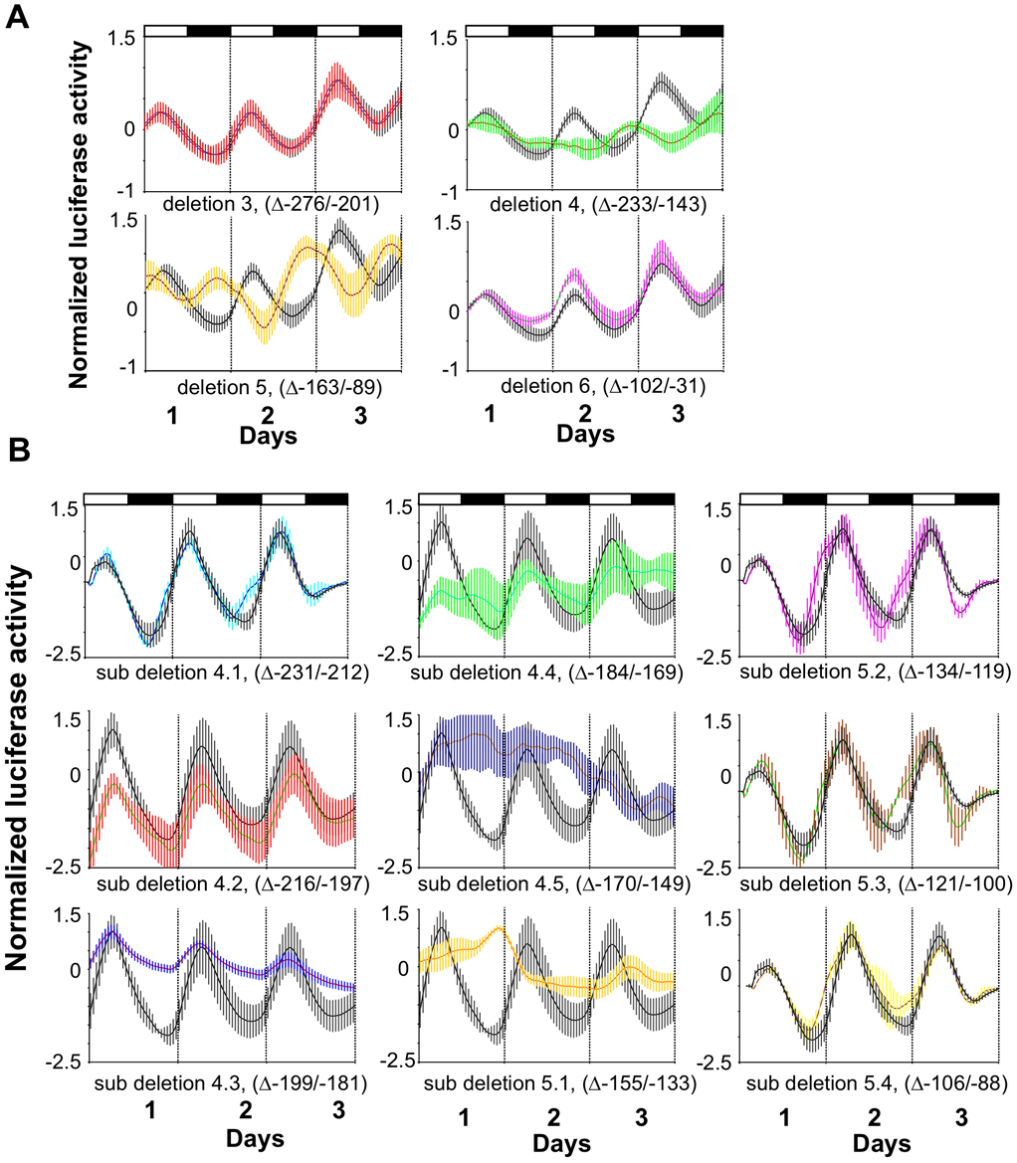
Identification of a LRM. (A) A series of partially overlapping ∼75 bp deletions were generated using the wild-type −*0.43per2:luc* construct. PAC-2 cells stably transfected with this series of constructs were monitored under LD conditions. Relative bioluminescence is plotted on the *y*-axis and time (days) on the *x*-axis. For each point, error bars represent the SD. White/black bars show the light and dark periods, respectively. Luciferase activity driven by the −*0.43per2:luc* construct is represented by a black line and grey bars; luciferase activities driven by deletions constructs are represented by coloured traces and SD bars. (B) A series of 10–20 bp deletions were performed in the region encompassed by Deletions 4 and 5. Cells transfected with sub-deletion constructs 4.3, 4.4, 4.5, and 5.1 exhibited an impaired light induced rhythmicity delimiting the LRM to a 67 bp region located at position −134 to −200.

**Figure 6:**
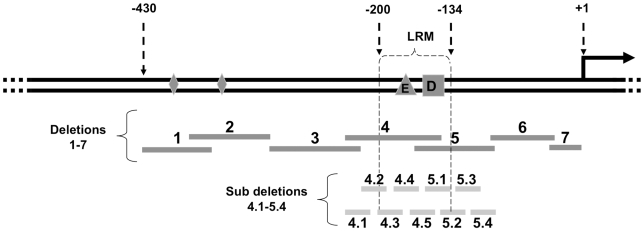
Schematic representation of the minimal 430 bp *per2* promoter and deletion constructs. The transcription start site is indicated (+1). A square denotes the D-box; a triangle denotes the E-box; diamonds represent the position of two non-canonical E-boxes. Deletions 1 to 7 are indicated as dark grey bars. Sub-deletions 4.1 to 5.4 are represented as light grey shorter bars.

To define more accurately the light responsive *cis*-regulatory sequences, a series of deletions of the *per2* promoter was prepared. First, a series of constructs carrying partially overlapping deletions within the −*0.43per2:Luc* wild-type construct was generated. Deletion constructs, stably transfected in PAC-2 cells, were then tested for light-regulated expression. Cells were exposed to LD conditions and the profiles of bioluminescence were compared with those of cells transfected using the wild-type −*0.43per2:Luc* construct (Figures 5 and 6). In both Deletion 4 and Deletion 5, the characteristic robust increase in expression observed following “lights on” in the wild-type promoter was absent. In addition, in the case of Deletion 4 cycling expression was severely attenuated, while for Deletion 5 the phase of rhythmic expression was significantly shifted (Figure 5A, Table 1). The remaining deletions did not affect either light-induced expression or the phase of rhythmic expression. These results point to a region of 145 bp (−89 to −233) containing the elements that are necessary for light-responsiveness.

To better define the light-responsive region, a second series of ∼20 bp sub-deletions within this 145 bp region were prepared and tested, in the context of the wild-type 0.43 kb promoter. PAC-2 cells were stably transfected with the sub-deletion constructs and exposed to LD cycles. Sub-deletions 4.3, 4.4, 4.5, and 5.1 resulted in a loss or severe reduction of light-induced expression in LD cycle conditions, whereas other sub-deletions within the wild-type promoter failed to show this effect (Figures 5B and 6, Table 1). These results delimit the core of the light responsive region to a 67 bp segment (−134 to −200), which we have termed “light responsive module” (LRM).

### The LRM Is Sufficient to Drive Light-Dependent Rhythmic Expression

**Figure 7:**
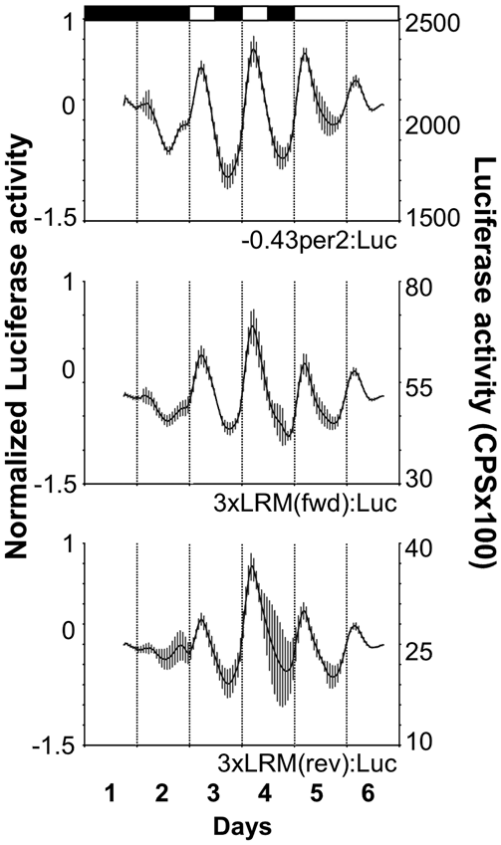
The LRM is sufficient to drive light-induced rhythmic expression. A region of 85 bp containing the LRM core with flanking regions (−218 to −132) was inserted three times in tandem into pLuc-MCS, an expression vector containing a synthetic TATA box only. Low background, non-cycling levels of expression of the empty vector were subtracted from the forward and reverse orientation heterologous promoter constructs. Normalized bioluminescence is plotted on the *y*-axis to the left and luciferase activity (counts per second) is plotted on the *y*-axis to the right. For each point, error bars represent the SD. White/black bars show the light and dark periods, respectively. The constructs containing the triple LRM insertion, in both forward and reverse orientation, drove light inducible rhythmic expression under LD and LL, indicating that the LRM is sufficient to drive light-induced expression in vivo.

An 87 bp region, containing the LRM core and flanking sequences (−218 to −132), was inserted, as three copies in tandem, in either the forward or reverse orientation into the TATA-box-containing pLuc-MCS plasmid (Stratagene) creating the *3xLRM(fwd):Luc* and *3xLRM(rev):Luc* constructs. Mean bioluminescence levels in cells transfected with either *3xLRM(fwd):Luc* or *3xLRM(rev):Luc* were lower (1%–2.5%) than those of the wild-type *per2* promoter. Nevertheless, both LRM constructs presented a light-driven expression pattern that was similar to that of the −*0.43per2:Luc* construct (Figure 7, Table 1), indicating that in addition to being necessary for light induction, the LRM also contains enhancer elements that are sufficient to direct light-driven rhythmic expression.

### The LRM Sequence and Function Are Evolutionarily Conserved

**Figure 8:**
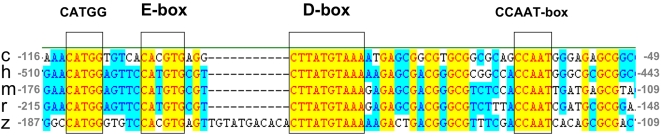
The LRM sequence is highly conserved among vertebrates. Multiple alignment of LRM-like sequences derived from chicken, human, mouse, rat, and zebrafish *per2* promoters. The conserved sequence is indicated by a yellow background. Studied elements; CATGG sequence, E-box, D-box, and CCAAT-box are bordered. Note that the distance between the E-box and D-box in the zebrafish *per2* promoter differs from that of the other species by 11 bp, which is equivalent to one DNA helical turn. Numbers on both ends of the LRMs indicate the distances from the putative transcription start sites.

Given the previous reports of elements of the *per2* promoter being evolutionarily conserved in mammalian species [20], we specifically examined whether the LRM core might itself represent an evolutionarily conserved regulatory element. Thus, the LRM core, with its flanking sequences (−184 to −120), was compared with the *per2* promoter sequences of chicken, mice, rat, and human (Figure 8). Interestingly, in all *per2* promoters, a region proximal to the transcription start site that exhibits high sequence identity (60%–63%) with the LRM was identified. Three sequences of interest are present within this conserved region of the *per2* promoter. These include (1) an E-box CAYGTG (where Y is a pyrimidine), known to mediate the activity of the positively acting clock component proteins [21], and (2) the sequence CTTATGTAAA that is perfectly conserved among the *per2* promoters and where 8/10 bases match the consensus D-box RT(G/T)AYGTAAY (where R is a purine). The D-box is the binding site for bZIP transcription factors of the proline and acid amino acid-rich (PAR) subfamily (DBP; TEF and HLF) and E4BP4. These transcription factors have been implicated in light-regulated phase shifting of the clock and in clock output pathways [22],[23]. (3) A CCAAT box known to play a role as a basal promoter element in polII transcribed genes [24].

**Figure 9:**
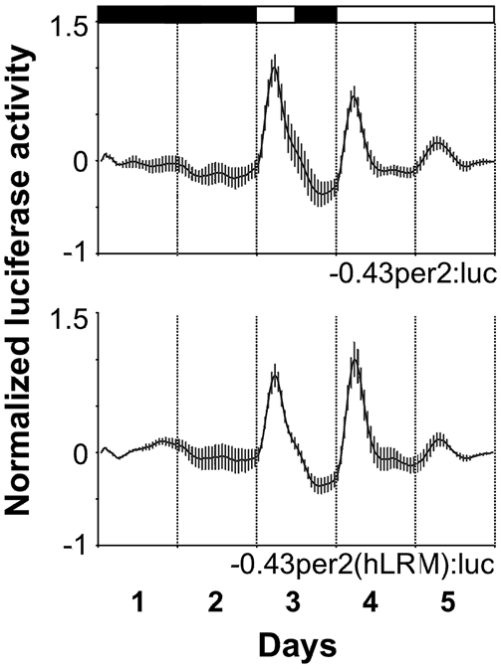
The hLRM is functionally conserved in the context of zebrafish cells. A fragment of 55 bp containing the core LRM within the −*0.43per2:luc* construct was replaced by the corresponding putative human LRM (hLRM), thus creating the −*0.43per2(hLRM):luc* construct. Relative bioluminescence is plotted on the *y*-axis and time (days) on the *x*-axis. For each point, error bars represent the SD. White/black bars show the light and dark periods, respectively. Remarkably, the hLRM-containing construct drove a similar expression profile as the wild-type construct implying that the LRM function is conserved throughout evolution.

Given the conservation of the LRM sequence between several vertebrate species, we questioned whether the functionality of LRM is also evolutionarily conserved. A fragment of 55 bp within −*0.43per2:Luc*, containing the core LRM (including the E-and D-boxes), was replaced by the corresponding 44 bp region of the human *per2* promoter (hLRM). PAC-2 cells were stably transfected with the resulting construct, −*0.43per2(hLRM):Luc*, and the expression profile was compared with that driven by the wild-type construct. Remarkably, expression profiles of the two constructs were similar under all the lighting conditions tested: LD, DD, and LL (Figure 9, Table 1). These results indicate that, in the context of zebrafish cells, the hLRM can mediate both light and circadian clock–directed expression.

### The LRM E- and D-boxes Are Essential for the Light Response of the *per2* Promoter

**Figure 10:**
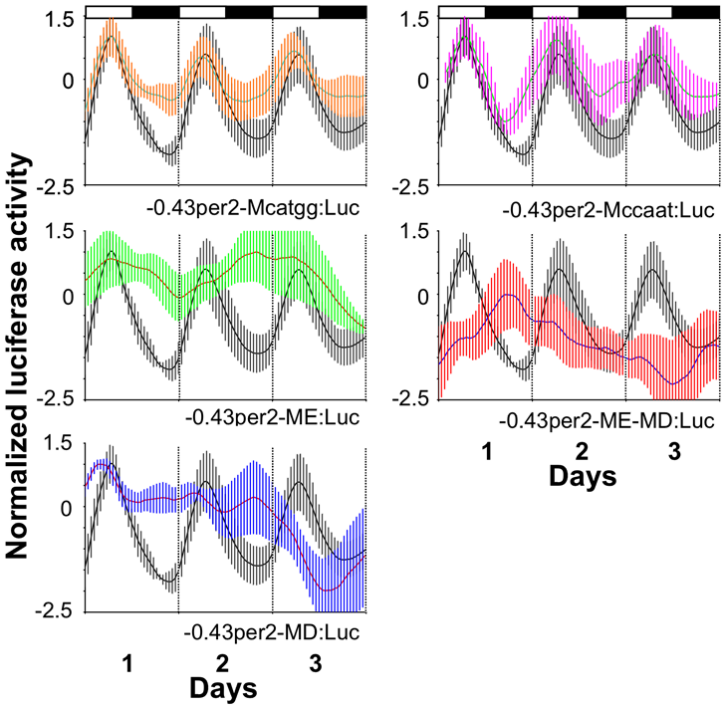
Point mutation analyses reveal that the LRM E-box and D-box elements are necessary for the light-induced rhythmic expression of *per2*. Cells were independently transfected with constructs that contain mutations of the following conserved motifs; CATGG sequence (−*0.43per2-Mcatgg:Luc*), E-box (−*0.43per2-ME:Luc*), D-box (−*0.43per2-MD:Luc*), CCAAT-box (−*0.43per2-Mccaat:Luc*), and both the E- and D-boxes (−*0.43per2-ME-MD:Luc*) and then luciferase activity was monitored. Relative bioluminescence is plotted on the *y*-axis and time (days) on the *x*-axis. The activity of the wild-type −*0.43per2:luc* construct is represented by a black line and grey SD bars. Mutation constructs are represented by coloured lines and SD bars. White bars represent light phases during the subjective day and black bars represent the dark phase. Luciferase activity was monitored under LD cycles.

To determine if any of the evolutionarily conserved putative elements (E-box, D-box, CCAAT-box, as well as an additional conserved CATGG sequence) within the LRM are required for its function, each element was point-mutated and the resulting promoter/reporter constructs were independently tested in stably transfected PAC-2 cells. Cells were then exposed to LD cycles, luciferase activity was monitored, and the expression profile was compared with those of cells transfected using the wild-type −*0.43per2:Luc* construct (Figure 10, Table 1). While point mutations disrupting the CCAAT box and the CATGG sequence had no major effect on the expression pattern under LD conditions, mutations of the E-box and D-box, singly or in combination, had a major disruptive effect on light-responsiveness. These results indicate that the E-box and D-box are crucial for LRM function, i.e., light-induction and clock regulation of the *per2* promoter.

**Figure 11:**
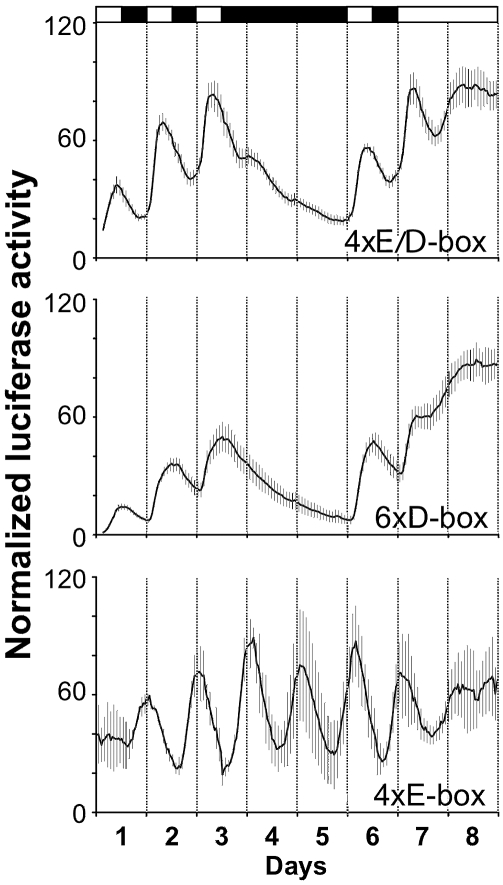
Adjacent E- and D-boxes confer LRM mediated transcription. PAC-2 cells were transiently transfected with a series of heterologous, E- and D-box-containing, luciferase reporter constructs. Transfected cells were then exposed to various lighting conditions as indicated by the black/white bars above the panels, and bioluminescence was monitored using a live-cell automated bioluminescence assay. Transfection results from heterologous constructs incorporating alternating E- and D-boxes (4xE/D-box) as well as multimerized D-boxes (6xD-box) and E-boxes-(4xE-box) are presented. Normalized luciferase activity (%) is plotted on the *y*-axis of each graph. Time (days) is plotted on the *x*-axis.

We next asked whether simply placing a canonical E-box adjacent to a canonical D-box element would be sufficient to confer the light-regulated pattern of expression observed for the *per2* promoter. To address this question, we constructed a heterologous promoter/reporter construct based on a canonical E-box (CACGTG) and a D-box (CTTATGTAAA) separated by 17 bp of non-*per2* LRM-derived sequence. Four tandem repeats of this artificial module were cloned upstream of a TATA box element, thereby driving transcription of a luciferase reporter gene (4xE/D-box). PAC-2 cells transfected with this construct were then exposed to various lighting regimes and luciferase activity was monitored (Figure 11, upper panel). Strikingly, a light-driven pattern of expression was observed that closely resembles that of the *per2* promoter, demonstrating the importance of E- and D-boxes in LRM function.

In order to dissect the individual contribution of the E- and D-boxes to the expression profile of the 4xE/D construct, we compared its profile with that of constructs containing either multimerized E-box (4xE-box, Figure 11, lower panel) or D-box (6xD-box, Figure 11, middle panel) elements. Consistent with previous results [17], the E-box directs rhythmic expression both in LD and free-running conditions (DD and LL) (Figure 11). Direct comparison of this expression pattern with that of the 4xE/D-box construct revealed clear differences: notably in the phase of the rhythms under LD conditions and the persistence of rhythmicity in DD. The D-box reporter, on the other hand, directs a robust light-driven pattern of expression. Under LD cycles, the bioluminescence levels increase 1 h after “lights on” and continue to increase until “lights off”, at which point they start to decline. Immediately following transfer to DD, levels decrease progressively with no cycling. Interestingly, however, upon transfer from LD to LL conditions, expression levels increase with a step-like profile hinting at some regulation by the endogenous circadian clock.

### E- and D-Box Binding Factors and Regulation of the LRM

**Figure 12:**
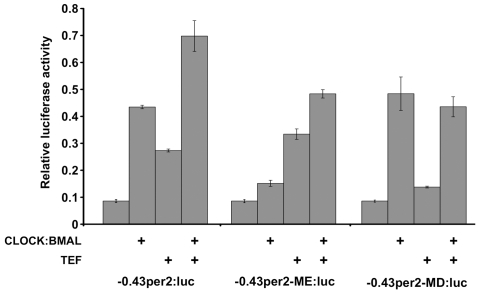
The LRM mediates TEF and CLOCK:BMAL activation in vitro. COS-7 cells were transfected with wild-type and mutated −*0.43per2:*luciferase constructs. Mutations include the LRM-E-box (−*0.43per2-ME:luc*) and the LRM-D-box (−*0.43per2-MD:luc*). The cells were co-transfected with combinations of expression vectors (indicated by +) of TEF and BMAL2/CLOCK1. Transcriptional activity is expressed as relative luciferase activity (mean±SE). Results are the mean of three independent experiments performed in triplicate. Statistical analysis was performed by three-way ANOVA. These results imply that both the BMAL/CLOCK complex and TEF contribute to the LRM activation.

What are the transcriptional control mechanisms targeting the E- and D-box enhancer elements? The E-box enhancer has been widely implicated as the regulatory target of the BMAL/CLOCK heterodimer. To confirm a role for CLOCK and BMAL in the regulation of the LRM via the canonical E-box, −*0.43per2:Luc* was co-transfected into mammalian COS-7 cells with either an empty vector (pcDNA3.1) or a mixture of zebrafish BMAL2 and CLOCK1 expression vectors (Figure 12). The COS-7 cell system was selected in order to test transcriptional activation in an endogenous “clock-free” environment [25]. Luciferase expression driven by the wild-type *per2* promoter was enhanced 5-fold (*p*<0.001 by three-way ANOVA) in the presence of BMAL/CLOCK (Figure 12). However, when the E-box was mutagenized (−*0.43per2ME:Luc*), the observed BMAL/CLOCK activation was 1.7-fold, significantly (*p*<0.001) lower than the activation of −*0.43per2:Luc*. These results suggest that the LRM E-box is capable of mediating transcriptional activation by BMAL/CLOCK.

**Figure 13:**
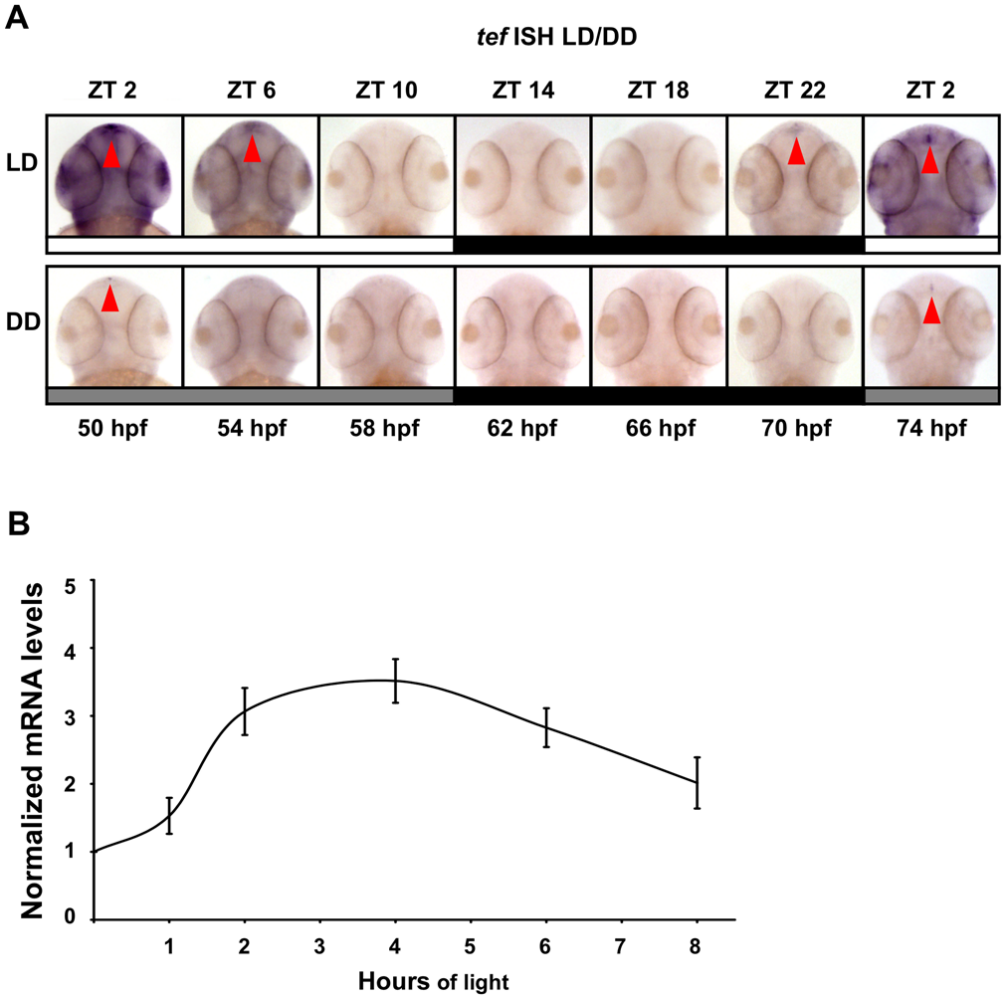
Temporal and spatial expression pattern of TEF under LD and DD cycles. (A) During the first 2 d of development, embryos were exposed to LD cycles. During the third and fourth days of development, embryos were kept under LD or under DD, sampled at 4 h intervals (50–74 hpf), and subjected to whole mount ISH for *tef* (Genebank Accession number U43671). White bars represent light phase, black bars represent dark phase, and gray bars represent subjective day (ZT, zeitgeber time). Red arrows indicate expression in the pineal gland. *Tef* is expressed throughout the body and cranial areas with augmented expression in the pineal gland and exhibits a circadian expression pattern with higher levels at the beginning of the subjective day (DD). Under LD, *tef* expression increases before lights on (ZT 2) and the amplitude of rhythmicity increases. (B) PAC-2 cells were maintained for 5 d in DD. Subsequently, total RNA was extracted from cells kept in darkness or exposed to light for different time periods (1, 2, 4, 6, 8 h). Quantification of *tef* mRNA levels was performed using qRT-PCR. The mRNA levels in each sample are expressed relative to the level of cells kept in DD. Values shown are the mean from three independent cell pools. Error bars represent SE. These results indicate that *tef* mRNA levels increase following exposure to light, peaking at 4 h of exposure. Statistical analysis was performed by one sample *t*-test. All light-treated samples showed significantly higher *tef* mRNA expression levels relative to DD controls.

Given the striking capacity of the D-box to confer light-driven expression in zebrafish, we chose to focus on how the function of D-box binding transcription factors responds to light. From studies in other vertebrates, bZIP transcription factors of the PAR domain subfamily and E4BP4 are known to bind to D-box elements. One candidate D-box activator, previously identified in zebrafish [26], is Thyrotroph Embryonic Factor (TEF). As a first step to evaluate whether TEF might be linked with light-regulated gene expression in zebrafish cells, we initially studied the effect of light on *tef* mRNA expression in whole embryos and PAC-2 cells. Whole mount ISH analysis was performed on 50–74 hpf embryos exposed to LD cycles or DD (Figure 13A). *Tef* expression appeared to be widely distributed throughout the body (unpublished data) and cranial areas with augmented expression in the pineal gland (Figure 13A). Interestingly, this pattern of expression is similar to that of *per2*, suggesting a possible link between these two genes (see also Figures 2 and 3). In addition, under LD cycles, *tef* levels increased rapidly following “lights on”, and in the pineal gland and in all other expression regions, reached maximum levels at ZT 2 and declined to undetectable levels by the end of the light phase (Figure 13A), indicating that *tef* expression is induced by light. An anticipatory behaviour of *tef* expression was observed: levels begin to increase before “lights on” (ZT22). Moreover, under DD *tef* mRNA levels exhibit low amplitude cycling, peaking at the beginning of the subjective day, indicating that *tef* expression is also regulated by the circadian oscillator.

To determine whether *tef* is light-induced in our experimental zebrafish cell system, qRT-PCR was performed upon cDNA prepared from PAC-2 cells that were kept in darkness or exposed to light for different time periods (Figure 13B). An increase in *tef* mRNA levels was observed when cells were exposed to light, reaching its highest levels after 4 h of exposure. Together, these results indicate that *tef* is up-regulated predominantly by light and partially by the circadian clock. Importantly, the phase of rhythmic *tef* expression precedes that of *per2*. These results provide a first clue for TEF being a regulatory factor contributing to the light-driven expression of *per2*.

We next tested whether TEF can activate the *per2* promoter through the LRM D-box. COS-7 cells were co-transfected with the *per2* promoter reporter constructs and a TEF expression vector (Figure 12). Wild-type *per2* promoter-driven luciferase expression was enhanced 3.3-fold while a non-significant increase (1.5 fold) was observed when the D-box was mutagenized (−*0.43per2MD:Luc*), suggesting that TEF acts as an activator of *per2* via the LRM D-box (Figure 12). Additionally, when cells were co-transfected with a combination of the BMAL/CLOCK complex along with TEF, an additive effect was observed indicating that both activators, BMAL/CLOCK and TEF, contribute simultaneously to the LRM activation through the E-box and D-box, respectively.

**Figure 14:**
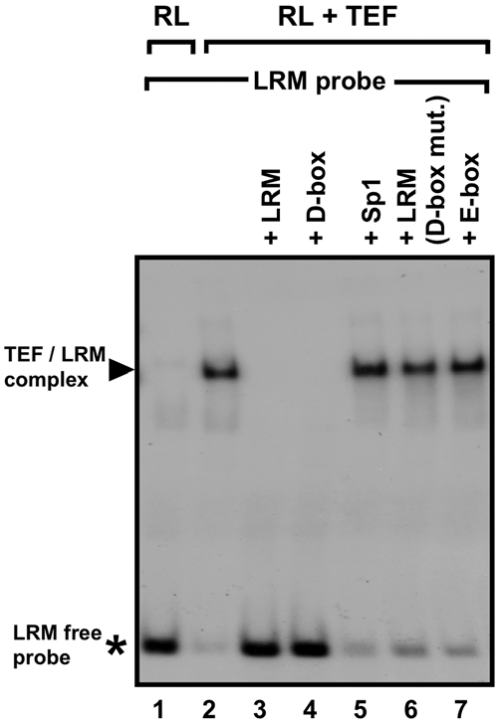
In vitro binding of TEF to the LRM D-box in an EMSA assay. A ^32^P-labeled LRM probe was incubated with in vitro–synthesized TEF protein, in the presence or absence of specific unlabeled competitor DNAs. Reticulocyte lysate (RL) alone was used as a control for unspecific binding between RL proteins and the probe (Lane 1). Then TEF without competitor (Lane 2), TEF with the LRM as competitor (Lane 3), TEF with the LRM D-box as competitor (Lane 4), TEF with an Sp1 site as competitor (Lane 5), TEF with an LRM probe carrying a mutated D-box as competitor (Lane 6), and TEF with the LRM E-box as competitor (Lane 7). Black arrow indicates the TEF/LRM complex. An asterisk indicates the free LRM probe. These results indicate that TEF binds to the LRM-D-box.

Aiming to test the ability of TEF to bind the LRM D-box sequences, electrophoretic mobility shift assays (EMSAs) were conducted using reticulocyte lysate-synthesized TEF. A radioactively labelled 50 bp DNA fragment of the LRM sequence was used as a DNA binding probe. Incubation of the LRM probe with in vitro synthesized TEF formed a single DNA-protein complex (Figure 14, Lane 2). To verify that TEF binds specifically to the D-box element within the LRM, unlabeled double-stranded oligonucleotides were used as competitors. While the LRM (Lane 3) or D-box (Lane 4) acted as effective competitors, an LRM probe containing a mutated D-box (Lane 6), as well as a SP1 (Lane 5) and a single E-box (Lane 7) binding site, was unable to compete. Together our results indicate that TEF is able to specifically bind the D-box element in the LRM core sequence and thereby activate *per2* expression.

### *Tef*: A Light-Regulated D-Box-Binding Transcriptional Activator of *per2*

**Figure 15:**
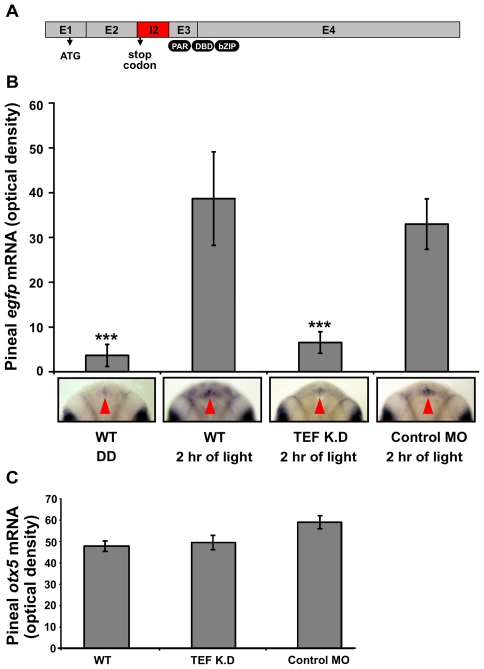
TEF is required for the light-induced *per2* promoter activation in vivo. Transgenic *Tg(*−*0.43per2:EGFP)tlv1* embryos were injected with *tef*-MO or control MO. Injected and uninjected embryos were entrained for two LD cycles. At the beginning of the third day of development, embryos were either exposed to light or kept in darkness. After 2 h (ZT 2) embryos were fixed and subjected to whole mount ISH for *egfp* or *otx5* mRNA. (A) *Tef* (E2I2) MO injection altered *tef* mRNA splicing. Sequence analysis of the PCR products indicate that *tef* (E2I2) MO caused the integration of the 226 nucleotides of intron 2, which creates a premature stop-codon (at position +1,303), generating a truncated, non-functional protein lacking the proline and acidic amino acid-rich region (PAR), DNA binding domain (DBD), and the basic leucine zipper domain (bZIP). Grey boxes represent exons, and a red box represents the integrated intron 2. Translation start site and integrated stop codon are marked by arrows. (B) Signal intensities, determined using ImageJ software, revealed a ∼10-fold increase in pineal *egfp* mRNA levels after a 2 h light pulse in uninjected (*n* = 20, *p*<0.001) and control MO injected (*n* = 16, *p*<0.001) embryos (see also Figure 3). *Tef*-MO-injected embryos (*n* = 22) showed no significant increase in pineal signal after a 2 h light pulse suggesting that *tef* is required for the light-induced expression driven by the −*0.43per2* promoter. Error bars represent SE. Dorsal views of representative whole mounts are shown below. Red arrows indicate the location of the pineal gland. (C) Pineal *zotx5* mRNA levels of *tef*-MO injected, control-MO-injected, and uninjected transgenic embryos were similar, indicating that the injected MOs did not affect the development of the pineal gland. Statistical analysis was performed by Kruskal-Wallis test followed by Mann-Whitney test.

In order to more directly test whether TEF contributes to light-induced expression of *per2* in vivo, we adopted a knock-down strategy. Morpholino-modified antisense oligonucleotides (MOs) corresponding to *tef* were injected into transgenic *Tg(*−*0.43per2:EGFP)tlv1* embryos. These morpholino oligonucleotides blocked the splicing of the second intron of the *tef* gene and thereby introduced a premature stop codon into the *tef* mRNA (Figure 15A). Injected embryos were then entrained to two LD cycles. At the beginning of the third day of development, the embryos were either exposed to 2 h of light or kept in darkness. Embryos were then fixed and *egfp* mRNA expression was assayed by whole mount ISH. In addition, the efficiency of *tef* (E2I2) MO directed against the splice site was evaluated by RT-PCR (unpublished data). Remarkably, knock-down of *tef* completely abolished the light-induced expression driven by the *per2* promoter (Figure 15B) but had no effect on the development or morphology of the pineal gland, as indicated by the normal expression pattern of *otx5* (Figure 15C). By contrast, injection of a control MO had no effect on the light-induced expression of the *per2* promoter (Figure 15B). These results indicate that TEF is involved and required in the light-induced pathway of *per2*.

## Discussion

In the last two decades, a great deal has been learned about the components and molecular organization of the circadian clock. However, the pathways by which the circadian oscillator is synchronized by light are still not fully understood. By focusing on the mechanisms whereby light induces the transcription of *per2*, this study aimed to identify regulatory elements that mediate light induction using the zebrafish as a model. Our in vivo analyses have defined the minimal *per2* promoter fragment that is light induced both in transgenic embryos and also in cell lines. In addition to the robust effect of light on *per2* expression, experiments under LD and LL conditions revealed that *per2* expression is also controlled by a circadian oscillator. An LRM within the promoter was identified and shown to be both necessary and sufficient for light-induced expression and clock regulation. We show that the zebrafish LRM is evolutionarily conserved and indeed can be functionally replaced by the LRM from the human *per2* gene. Our mutational analyses indicate that adjacent D-box and E-box elements underlie the LRM function. We demonstrate that the D-box contributes to light-driven transcriptional control while the E-box mediates circadian clock control. The E-box is the target of the BMAL/CLOCK heterodimer, the positive components of the core circadian oscillator, while the bZIP PAR domain transcriptional activator TEF contributes to light-dependent regulation of the D-box.

### D-Boxes: Light-Responsive Enhancer Elements in Zebrafish

Our study of the light-regulated *per2* promoter has revealed that D-box enhancers direct a light-driven expression pattern in zebrafish cells. This result is important for our general understanding of how transcriptional regulatory mechanisms have evolved in the vertebrate circadian clock. In mouse, D-box binding factors include bZIP transcriptional activators of the PAR domain subfamily (DBP, TEF, HLF) and the E4BP4 repressor. Detailed reverse genetics studies clearly point out that the D-box mediates circadian clock output [27]. CLOCK and BMAL regulate expression of DBP via E-box enhancers in its promoter and thereby drive a high amplitude circadian rhythm of DBP expression that in turn confers circadian clock control on the expression of DBP target genes [28]. Consistently, in mammalian cell culture assays, a D-box reporter construct similar to the 6xD-box described here displays a characteristic clock-regulated rhythmic expression profile [29]. Thus our findings in the zebrafish model, linking D-box regulation predominantly with the light input pathway, points to plasticity in the precise role of this particular gene expression regulatory pathway during the course of evolution. The ancestral genome duplication that occurred during the evolution of the teleost lineage and the resulting extra copies of many zebrafish genes including clock genes implies that there might also be extra copies of D-box binding factors. Thus while our data clearly implicate *tef* in light-dependent regulation of *per2*, it will be fascinating to identify and explore the role of additional teleost D-box regulatory factors and compare their function with the mammalian counterparts.

### *Per2*: Light-Dependent Clock Regulation

Previous studies in zebrafish have concluded that transcription of the *per2* gene is exclusively light regulated and is not under circadian clock control [9],[18],[30]. In the current study, we have used a live cell bioluminescence assay of stably transfected zebrafish cell lines that provides us with a high-resolution, real-time image of changes in *per2* promoter-driven expression. Using this assay we find that the rhythmic activity of the zebrafish *per2* promoter under LD exhibits anticipatory behaviour: expression levels start decreasing before lights off and begin to rise before lights on. Moreover, a circadian rhythm of expression is also observed under LL. Clues as to the origin of this clock-regulated expression come from our studies of the regulation by the individual E- and D-box elements. Both elements direct a circadian rhythm of expression under LL conditions. This result is consistent with previous studies demonstrating that the zebrafish circadian clock continues to direct circadian rhythms of gene expression under LL conditions [17],[31]. However, it remains unclear whether clock regulation by the D-boxes in LL represents a direct effect of the core clock machinery or might reflect an indirect effect, for example on the sensitivity of the light input pathway. Taken together, these results clearly implicate the circadian oscillator in the regulation of *per2*. Nevertheless, in contrast to purely E-box-regulated expression, rhythmic expression of *per2* is absent under DD, thus pointing to a hierarchic control, where light enables the circadian clock regulation of the *per2* promoter.

### The LRM Regulatory Mechanism

Recent studies in mouse and chicken addressing the mechanism underlying rhythmic *per2* transcription have implicated three signalling pathways. One is the cAMP pathway that may stimulate *per2* expression under certain conditions [32],[33]. A second pathway involves CLOCK/BMAL that activate the expression of mouse *per2* via a non-canonical E-box [34]. A third pathway involves E4BP4 that acts as a suppressor of *per2* promoter activity in both chicken and mouse through binding to a D-box [20],[22]. This possibly reflects the existence of a feedback between clock outputs and the core clock mechanism itself that is mediated by D-box binding factors [27]. In the mouse and chicken models, the elements that mediate these activities are located outside of the conserved LRM and also separated from each other in terms of linear DNA sequence. Thus, the E-box is situated in the proximal promoter region of the mouse *per2* gene, and the identified D-box is situated in a distal promoter region and first intron of the chicken and mouse *per2* genes, respectively. The presence of E-box and D-box sequences within the conserved LRM was mentioned in the mouse and chicken studies, but this D-box was not shown to bind E4BP4.

**Figure 16:**
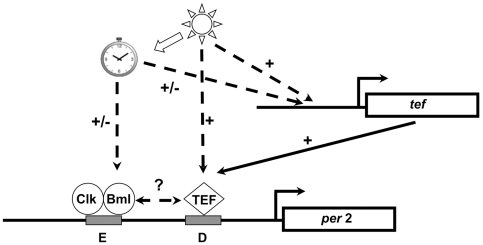
Light and clock-directed regulation of the LRM. A model is proposed where the *per2* LRM E- and D-boxes represent a convergence point for both clock and light-driven transcription control mechanisms. The core clock mechanism regulates the E-box enhancer (E) via CLOCK (Clk) and BMAL (Bml) heterodimer, while light exposure predominantly drives expression from the D-box by regulation of the D-box binding factor, TEF. In addition to resetting the phase of the clock mechanism (block arrow), light exposure induces transcription via TEF in two possible ways. In one mechanism, light exposure triggers post-translational modification of existing TEF protein. In another mechanism, light induces the *de novo* expression of the *tef* gene that in turn contributes to an increase in transcriptional activation by the D-box. Interaction between the E- and D-box binding factors ultimately defines the precise kinetics of light-regulated *per2* expression.

Importantly, the findings of the chicken and mouse studies concern the effects of light perceived by dedicated photoreceptor cells and then relayed indirectly to responsive cells. However, the findings of the current study reveal the effects of direct exposure of cells to light, made possible by the use of zebrafish cell lines. Although the experimental systems that were used in the chicken and mouse studies failed to assign functionality to the LRM and its constituting elements, the remarkable evolutionary conservation of the LRM and the fact that the human LRM sequence functioned as a light-regulated enhancer in the context of the zebrafish cells suggests that mammalian LRMs may well also play an important regulatory role in entrainment. Alternatively, during evolution, ancestral light-responsive mechanisms may have been subverted to respond to signals other than light. It would therefore be interesting to determine whether TEF binds the D-box in LRMs of chicken and mice. Our data demonstrate that proximally spaced D- and E-boxes are sufficient to confer the light- and clock-regulated expression pattern as shown by the *per2* promoter. This expression pattern represents a combination of the regulation exerted by the two separate enhancers. It is likely that the E-box confers the anticipatory rise in expression prior to “lights on” and the decline in expression prior to “lights off” under LD conditions and the low amplitude cycle observed during the first few cycles after transfer to LL. The D-box confers the strong up-regulation of expression after “lights on” and the rapid attenuation of expression in DD conditions as well as contributing to rhythmic expression under LL conditions. We thus propose the following model for the LRM function and regulation by the E-box and D-box binding factors (Figure 16). Interaction between the E- and D-box enhancer elements confers a hierarchic control in which light enables circadian clock regulation. The D-box represents the binding site for a family of bZIP transcription factors; the current results implicate the light-inducible D-box-binding factor TEF as a key player in activating *per2* expression. Thus in the proposed model, at the onset of the day, light exposure activates TEF and thereby results in a D-box-mediated transcriptional activation of *per2*. D-box regulatory factors in turn interact with the CLOCK/BMAL heterodimer through the proximal E-box. The clock-driven daily changes in CLOCK/BMAL transcriptional activation contribute to the elevation of *per2* mRNA levels in the beginning of the light phase and result in the anticipatory reduction of *per2* during the light phase. Clock-regulated changes in *tef* gene expression may further contribute to the rhythms of *per2* expression observed under LD and LL conditions. Importantly, previous results have demonstrated that upon blocking of de novo protein synthesis, *per2* light-induced expression persists although with a delay in the arrival at peak expression levels [35],[36]. This is consistent with TEF already being expressed in the cell prior to light exposure and that light may also serve to activate TEF via post-translational mechanisms.

### A General Mechanism for Light-Regulated Gene Expression in Zebrafish?

In the intact animal, *per2* expression increases ubiquitously following light exposure and is mainly enhanced in the pineal gland. Furthermore, *tef* mRNA appears to be widely expressed with high levels in the pineal gland, where its light-induced expression slightly precedes that of *per2*. The proposed mechanism (Figure 16) that is based on experiments in cell lines that represent peripheral clock-containing tissues may therefore be applicable to the increased ubiquitous expression. The enhanced expression in the pineal gland is in accordance with the fact that it constitutes a dedicated photoreceptive organ and a central clock tissue in fish. The relevance of the proposed mechanism to light entrainment of the central clock in the pineal gland remains to be determined.

Several other genes have been shown to be light inducible in zebrafish cells and tissues. These include the clock gene *cry1a*, suggesting that light may entrain the circadian clock mechanism by regulating the transcription of multiple clock gene elements. Interestingly however, unlike the case of *per2*, treatment with the protein synthesis inhibitor, cycloheximide, completely abolishes light-induced *cry1a* expression [35]. This result implies that there may be more than one mechanism whereby light regulates gene expression. Other non-clock-related genes such as *6-4 DNA photolyase* have also been demonstrated to be inducible by acute exposure of zebrafish cells to light [37]. The induction of genes involved in the repair of UV damaged DNA by visible wavelengths of light seems likely to represent part of an adaptive strategy to optimize the cellular response of DNA damage repair during exposure to sunlight when it is most needed. It will therefore be fascinating to determine whether the LRM sequences or even D-box elements also play a more general role in light-dependent changes in zebrafish cell physiology.

In summary, our study has revealed a novel mechanism that combines light-induced and clock-regulated transcriptional control. The proposed mechanism may lead to a better understanding of the phenomenon of gated light entrainment of the circadian clock and possibly other general photic cellular responses.

## Materials and Methods

### Fish Maintenance

Adult zebrafish were raised in a recirculation water system under 12 : 12 h LD cycles at 28°C and fed twice a day. To produce embryos, male and female zebrafish were paired in the evening, and spawning occurred the next day within 1 h after “lights on”. Embryos were placed in 10 cm Petri dishes with egg water containing methylene blue (0.3 ppm) and raised in a light-controlled incubator at 28°C (light intensity, 12 W/m2). To prevent pigmentation, the fish water was supplemented with 0.2 mM phenylthiourea during the first day of development.

### DNA Constructs: Constructs for In Vivo Analyses

#### −1.8per2:EGFP

The *per2* promoter was subcloned into pEGFP-1 (Clontech) upstream of the EGFP reporter gene. A fragment containing 1,813 bp of the 5′ flanking region and 164 bp of the 5′ untranslated region (UTR) of the *per2* gene (accession number FJ435339) was PCR amplified from genomic DNA using specific primers PER2PRO(1977)F (incorporating a *Sal*I restriction site [5′–cgcgtcgacatcatttcccagtgcttagtggcagatg–3′] and designed according to the available genomic sequence), and PER2EX1R (containing a *Bam*HI restriction site [5′–cgcggatccctgacaacttcagcaaatcttctttttcgc–3′] and based upon genomic and the 5′ UTR sequence, accession number FJ435338). The PCR product was double-digested with *Sal*I and *Bam*HI and ligated into *Bam*HI/*Sal*I-digested pEGFP-1.

#### −0.9per2:EGFP

A fragment containing 915 bp of the 5′ flanking region and 164 bp of the 5′ UTR of *per2* was subcloned into pEGFP-1 as described for −*1.8per2:EGFP* except for the use of a different forward primer, PER2PRO(1079)F, that also contained a *Sal*I restriction site (5′–cgcgtcgactccagagactgcaacccactcatattgg–3′).

#### −0.43per2:EGFP

A fragment containing 431 bp of the 5′ flanking region and 164 bp of the 5′ UTR of *per2* was subcloned into pEGFP-1 as described for −*1.8per2:EGFP* except for the use of a different forward primer PER2PRO(595)F containing a *Sal*I restriction site (5′–cgcgtcgacaacctattggatcacttcgaggcatcac–3′).

#### Tg(−0.43per2:EGFP)

This construct was prepared as described for −*0.43per2:EGFP* except for subcloning into *Bam*HI/*Xho*I-digested pT2ALR150G [38]. This construct was later used for the preparation of transgenic lines utilizing the *Tol2* system (see below) [38].

### Constructs for In Vitro Analyses

#### −1.7per2:Luc

A fragment containing 1,700 bp of the 5′ flanking region of *per2* was subcloned into pGL3basic (Promega) upstream to the luciferase gene. A fragment containing 1,571 bp of the 5′ flanking region and 129 bp of the 5′ UTR of the *per2* gene was PCR amplified using the Universal GenomeWalker Kit (Clontech) according to the manufacturer's instructions, using the specific primer PER25UTREX1R (5′– AGCCTTGCTTCAAAACAGGTCTCAGTG –3′) in combination with the AP1 primer (Clontech). The resulting product was subcloned into the pGEM–T easy vector (Promega) and sequenced. The 1,700 bp fragment was then double digested with *Not*I, blunt ended by Klenow treatment (New England Biolabs), and then ligated into *Sma*1-digested pGL3.

#### −0.43per2:Luc

The *per2* minimal promoter was subcloned into pGL3basic upstream of the luciferase reporter gene. A fragment containing 431 bp of the 5′ flanking region and 164 bp of 5′ UTR of the *per2* gene was PCR amplified using *−0.43per2:EGFP* as a template and a set of specific primers: PER2PRO(595)bF, containing a *Nhe*I restriction site (5′–cgcgctagcaacctattggatcacttcgaggcatcac–3′), and PER2EX1bR, containing a *Bgl*II restriction site. The PCR product was double-digested with *Nhe*I and *Bgl*II and ligated into *Nhe*I/*Bgl*II-digested AVP-pGL3 [39] to replace the AVP sequence.

### Deletion Mutations

#### −0.43per2(Δ−437/−393):Luc (Deletion 1)

A mutation creating a *Nhe*I recognition site located at position −393 in −*0.43per2:Luc* was accomplished using a set of two complementary primers: Nhe1Del1F (5′–cccgcacgtccatgctagctattgtaaaagc-3′) and Nhe1Del1R (5′–gcttttacaatagctagcatggacgtgcggg–3′), which incorporated the desired mutation using the QuikChange Site-directed mutagenesis kit (Stratagene) as instructed by the manufacturer. The construct was then digested using *Nhe*I, and re-ligated resulting in a 45 bp deletion of the 5′ end of the 430 bp promoter.

All subsequent deletion, sub-deletion, and point mutation constructs were also generated using the QuikChange Site-directed mutagenesis kit.

#### −0.43per2(Δ−392/−288):Luc (Deletion 2)

The deletion of a 105 bp region, partially overlapping Deletion 1, was obtained using a set of two primers, located 105 bp apart Del2F (5′–gcatggacgtgcgggatgcc–3′, corresponding to nt −287 to −268) and Del2R (5′–cggcttccccggaccaacca–3′, corresponding to nt −412 to −393)

#### −0.43per2(Δ−276/−201):Luc (Deletion 3)

A deletion of 76 bp was generated using primers Del3F (5′–cggggaagccgagtacgctgtagtgt–3′, corresponding to nt −200 to −179) and Del3R (5′–gcactccgtcactggccatggg–3′, corresponding to nt −302 to −277).

#### −0.43per2(Δ−233/−143):Luc (Deletion 4)

A deletion of 91 bp partially overlapping Deletion 3 was created using primers Del4F (5′–gactgacgggcatttcgaccaatcac–3′, corresponding to nt −142 to −117) and Del4R (5′–ctggtgaatgggacgctgacgagg–3′, corresponding to nt −234 to −211).

#### −0.43per2(Δ−163/−89):Luc (Deletion 5)

A deletion of 75 bp partially overlapping Deletion 4 was created using primers Del5F (5′–cgcatacaaatccgcaggatttaccca–3′, corresponding to nt −88 to −62) and Del5R (5′–caactcacgtggtcacccatggcc–3′, corresponding to nt −164 to −187).

#### −0.43per2(Δ−102/−31):Luc (Deletion 6)

A deletion of 72 bp partially overlapping Deletion 5 was created using primers Del6F (5′–caggtcatgctgcgagttctggagatc–3′, corresponding to nt −30 to −4) and Del6R (5′–gtgagagtcgcgctgtgattggtcg–3′, corresponding to nt −127 to −103).

#### −0.43per2(Δ−37/−1):Luc) (Deletion 7

A deletion of 37 bp partially overlapping Deletion 6 and up to the transcription start site was created using primers Del7F (5′–ggcagcggtgttagagagcagtcaacg–3′, corresponding to nt +1 to +27) and Del7R (5′–actccgccctccgagggctcc–3′, corresponding to nt −58 to −38).

### Sub-Deletions

#### −0.43per2(Δ−231/−212):Luc (Deletion 4.1)

A deletion of 20 bp was created using primers Del4.1F (5′–tccatacgcagcfgcactccgtcac–3′, corresponding to nt −211 to −188) and Del4.1R (5′–gctggtgaatgggacgctgacgag–3′, corresponding to nt −255 to −232).

#### −0.43per2(Δ−216/−197):Luc (Deletion 4.2)

A deletion of 20 bp was created using primers Del4.2F (5′–ctccgtcactggccatgggtgacc–3′, corresponding to nt −196 to −173) and Del4.2R (5′–cagtagtaagagaatgctggtgaatgggacgc–3′, corresponding to nt −248 to −217).

#### −0.43per2(Δ−199/−181):Luc (Deletion 4.3)

A deletion of 19 bp was created using primers Del4.3F (5′–gggtgaccacgtgagttgtatgacacacttat–3′, corresponding to nt −180 to −149) and Del4.3R (5′–gctgcgtatggacaaatcagtagtaagagaatgc–3′, corresponding to nt −233 to −200).

#### −0.43per2(Δ−184/−169):Luc (Deletion 4.4)

A deletion of 16 bp was created using primers Del4.4F (5′–gagttgtatgacacacttatgtaaaaagactgacggg–3′, corresponding to nt −168 to −132) and Del4.4R (5′–ccagtgacggagtgcgctgcg–3′, corresponding to nt −205 to −185).

#### −0.43per2(Δ−170/−149):Luc (Deletion 4.5)

A deletion of 22 bp was created using primers Del4.5F (5′–gtaaaaagactgacgggcatttcgacc–3′, corresponding to nt −148 to −122) and Del4.5R (5′–gtggtcacccatggccagtgacg–3′, corresponding to nt −193 to −171).

#### −0.43per2(Δ−155/−133):Luc (Deletion 5.1)

A deletion of 23 bp was created using primers Del5.1F (5′–gcatttcgaccaatcacagcgcg–3′, corresponding to nt −132 to −110) and Del5.1R (5′–tgtcatacaactcacgtggtcacccatg–3′, corresponding to nt −183 to −156).

#### −0.43per2(Δ−134/−119):Luc (Deletion 5.2)

A deletion of 16 bp was created using primers Del5.2F (5′–cacagcgcgactctcacatttccgtatt–3′, corresponding to nt −173 to −135) and Del5.2R (5′–gtcagtctttttacataagtgtgtcatacaactcacgtg–3′, corresponding to nt −118 to −91).

#### −0.43per2(Δ−121/−100):Luc (Deletion 5.3)

A deletion of 22 bp was created using primers Del5.3F (5′–ttccgtattttacgcatacaaatccgcagg–3′, corresponding to nt −99 to −70) and Del5.3R (5′–ggtcgaaatgcccgtcagtctttttaca–3′, corresponding to nt −149 to −122).

#### −0.43per2(Δ−106/−88):Luc (Deletion 5.4)

A deletion of 20 bp was created using primers Del5.4F (5′–cgcatacaaatccgcaggatttaccca–3′, corresponding to nt −87 to −61) and Del5.4R (5′–gagtcgcgctgtgattggtcgaaatg–3′, corresponding to nt −132 to −107).

### Point Mutations

#### −0.43per2-ME:Luc

The E-box located at position −174 to −169 was mutated (from CACGTG to CTCGAG). Two complementary primers ME1F (5′–ggccatgggtgacctcgagagttgtatgacacac–3′) and ME1R (5′–gtgtgtcatacaactctcgaggtcacccatggcc–3′) containing the desired mutation were used to introduce the mutations into the −*0.43per2:Luc* construct.

#### −0.43per2-MD:Luc

The putative D-box located at position −154 to −145 within the −*0.43per2:Luc* was mutated. A complementary set of primers ME4BP4F (5′–ccacgtgagttgtatgacacactcctctagaaagactgacgggc–3′) and ME4BP4R (5′–gcccgtcagtctttctagaggagtgtgtcatacaactcacgtgg–5′) containing the desired mutation (CTTATGTAAA to CTCCTCTAGA) were used to disrupt the D-box in the −*0.43per2:Luc* construct.

#### −0.43per2-ME-MD:Luc

The primers used to prepare the −*0.43per2-MD:Luc* construct were also used to introduce the mutations into −*0.43per2-ME:Luc*, thus creating the double mutated construct.

#### −0.43per2-Mcatgg:Luc

A conserved sequence located within the LRM at position −184 to −180 was mutated (CATGG to GGAGC). Two complementary primers McatggF (5′–gcgcactccgtcactggcggagcgtgaccacgtgag–3′) and McatggR (5′–ctcacgtggtcacgctccgccagtgacggagtgcgc–3′) containing the desired mutation were used to introduce the mutations into the −*0.43per2:Luc*.

### Recombinant Promoter Constructs

#### 3xLRM(fwd):Luc and 3xLRM(rev):Luc

Three copies of the 87 bp sequence containing the LRM core and flanking sequences (corresponding to nt −218 to −132) were inserted in tandem into pLuc-MCS (Stratagene). Using the −*0.43per2:Luc* plasmid as a template, the 87 bp fragment was PCR amplified using primers XmaI(LRM)R (5′–cgccccggggcccgtcagtctttttacataagtgt–3′) and XhoI(LRM)F (5′–cgcctcgagatttgtccatacgcagcgca–3′). The product was subcloned into pGEM-T-easy creating the *LRM-pGEM* construct. The same fragment was amplified using XhoI(LRM)F and XhoI(LRM)R (5′–cgcctcgaggcccgtcagtctttttacataagtgt–3′). The PCR product was digested with *Xho*I and two additional copies were ligated into *XhoI* digested *LRM-pGEM*. The fragment containing three copies of the LRM was digested with *Eco*RI and ligated into *Eco*RI-digested pLuc-MCS, resulting in clones with inserts in both the forward and reverse orientation.

#### −0.43per2(hLRM):Luc

The zebrafish 55 bp LRM was deleted from the −*0.43per2:Luc* using primers zLRMdelF (5′–ccaatcacagcgcgactctcacatttcc–3′, corresponding to nt −123 to −96) and zLRMdelR (5′–ccatggccagtgacggagtgcgct–3′, corresponding to nt −202 to −179). The product was blunt-end ligated to annealed oligos corresponding to the human 44 bp LRM (5′–agttccatgtgcgtcttatgtaaaaagagcgacgggcgcggcca–3′), thus replacing the zebrafish LRM with the human LRM.

### Heterologous Promoter Reporter Constructs

#### 4xE-box

This E-box reporter construct contains four copies of the E-box sequence from the *period4* promoter of zebrafish cloned into the vector pLucMCS. The precise sequence and construction details have been reported elsewhere [17].

#### 6xD-box

Six copies of the sequence 5′-tgcgtcttatgtaaaaagag-3′ (D-box from the *hper2* gene promoter, position −488 [40]) were cloned into pLucMCS to generate 6xD-box.

#### 4xE/D-box

Oligonucleotides consisting of four copies of the sequence 5′-gaagcacgtgtactcgaaagagtgcgtcttatgtaaaaagagtgcg-3′ (an E-box sequence from the *period4* promoter [position −7] and a D-box from the *hper2* promoter [position −488]) were cloned into pLucMCS to generate 4xE/D-box.

### Expression Vectors

#### pcDNA3.1-TEF

The coding sequence of *tef* (Accession number U43671 [26]) was subcloned into pcDNA3.1 (Version A, Invitrogen). A 905 bp fragment was PCR amplified from PAC-2 cells cDNA library, using specific primers TEFa F (5′-gcgggtaccatgaagcctatttccatcacgatgg-3′) and TEFa R (5′-gcggaattcttacagcgctccgtatttggcttc-3′), which contain *Kpn*I and *Eco*RI restriction sites, respectively. The PCR product was double-digested with *Kpn*I and *Eco*RI and ligated into *Kpn*I/*Eco*RI digested pcDNA3.1 (Version A). Expression vectors for zebrafish CLOCK1 and BMAL2 also based on pcDNA3.1 have been described previously [16].

### In Vivo Transient Expression Assay and Examination of Embryos

Transient expression assays of the EGFP-reporter constructs were performed by microinjection of zebrafish embryos as described previously [41],[42]. Following injections, embryos (∼300) were incubated in a 10 cm plastic dish at 28°C. Embryos were examined and graded during the light phase on Day 2–5 post-fertilization. Green fluorescence in live embryos was detected as previously described [41]. Since *per2* is ubiquitously expressed [18],[43], embryos were considered “positive” if fluorescence was detected anywhere in the embryo's body. Embryos were housed in 10 cm dishes (∼60 embryos per dish) for additional daily observation and validation until Day 5. Results are presented as percentage of EGFP expressing embryos.

### Preparation of Transgenic Fish

The transgenic line *Tg(–0.43per2:EGFP)tlv1* was generated using the *Tol2* system as described [19]. Plasmids were kindly provided by Koichi Kawakami. Briefly, transposase mRNA was synthesized in vitro using mMESSAGE mMACHINE SP6 Kit (Ambion Inc.). Approximately 1 nl of a DNA/RNA solution containing 25 ng/µl of *Tg(–0.43per2:EGFP)* circular DNA and 25 ng/µl transposase mRNA were injected into each fertilized egg. Founder (F0) fish were crossed and EGFP expressing progeny (F1) were raised to adulthood. F2 progeny from out crossed F1 fish were used.

### Morpholino Design and Injection

Gene knock-down experiments were performed using morpholino-modified antisense oligonucleotides (MO; Gene Tools): Gene Tools standard control MO (5′-ctcttacctcagttacaatttata-3′) and *Tef* (E2I2) MO (5′-agtgttctgttcttacagacctgat-3′), which was designed to target the exon 2-intron 2 boundary to interfere with splicing. MO injected embryos (2 nl, 1 mM) were incubated and fixed as described above. Efficiency of *tef* (E2I2) MO, directed against the splice site, was evaluated by RT-PCR. Uninjected, *tef* (E2I2) MO and control MO-injected embryos were entrained to two LD cycles and sampled at 50 hpf, after 2 h light exposure or darkness, and total RNA was extracted as previously described [44]. DNA fragments were then PCR-amplified by using primers directed to exons 1 and 4 of *tef* (TEFa F and TEFa R described above). PCR products were cut out from gel, purified, and sequenced.

### Whole Mount ISH Quantification and Statistical Analysis

mRNA expression was detected by whole mount ISH as described [18],[45]. Embryos/larvae exposed to different photic regimes were fixed overnight in 4% paraformaldehyde and stored in 100% methanol. Whole mount ISH was performed with a dioxygenin (DIG)-labelled probe at a concentration of 1 ng/µl. Detection and documentation of the signal was performed as described [18],[45]. The ISH signal, expressed as optical density, was quantified by using ImageJ software (National Institutes of Health, Bethesda, MD, USA) as described [18],[45]. Statistical differences in signal intensities between treatments were determined by one-way ANOVA or Kruskal-Wallis test followed by Mann-Whitney test. Results are expressed as mean total optical density ± standard error.

### In Vitro Transient Transfection Assays in COS-7 Cells

COS-7 cells were plated at a density of 3×10^4^ cells per well in a 24-well plate (Costar) and transfected 24 h later with a mixture containing Lipofectamine Plus (1.25/2.5 µl) reagents (Invitrogen), 10 ng of *−0.43per2:Luc*, *−0.43per2-ME:Luc* or *−0.43per2-MallE:Luc*, and 0.75 µg of a 1∶1∶1 expression vector mix of zebrafish TEF, BMAL2, and CLOCK1 [16] or empty vector pcDNA 3.1 (Invitrogen) in 50 µl of Vitacell Dulbecco's modified Eagle's medium (ATCC) without fetal bovine serum. On the following day, 0.5 ml of culture medium (Vitacell Dulbecco's modified Eagle's medium supplemented with 10% fetal bovine serum) was added to each well; cells were harvested 24 h later. Differences in transfection efficiency were controlled by measuring the enzyme activity generated by a co-transfected thymidine kinase promoter-driven *Renilla* luciferase (RL) plasmid (0.5 ng). Firefly and RL enzyme activities were measured using the Stop and Glo kit (Promega) following the manufacturer's instructions and relative luciferase activity was determined for each well. Results are the mean of three independent experiments, each performed in triplicate.

### In Vitro Transient Transfection Assays in PAC-2 Cells

PAC-2 cells were transfected using Fugene6™ transfection reagent according to the manufacturer's instructions with a 4∶1 ratio of FugeneHD (in µl)∶DNA(in µg) (Roche Diagnostics, FugeneHD) and subsequently incubated for 24 h at 25°C prior to the in vivo luciferase assay.

### Establishment of Stable PAC-2 Cell Lines

PAC-2 cells [10] were cultivated as previously described [10],[17],[46]. Cells were transfected with *Kpn*I-linearized luciferase reporter plasmids (listed above) and a neomycin resistance plasmid linearized with *Eco*RI [pcDNA3,1 His-Myc(A), (Invitrogen)] at a molar ratio of 7∶1. Electroporation was performed at 0.29 kV, 960 µF, by using a Gene Pulser apparatus (Bio-Rad). Three days later, Neomycin G-418 (Gibco BRL) was added at a final concentration of 800 ng/µl. During 1 mo of selection, the concentration was gradually reduced to 250 ng/µl, and 100–200 resistant colonies per transfection were obtained. Colonies were trypsinized and propagated as a single pool.

### Live Cell Luciferase Assay and Data Analysis

Live cell luciferase assays were performed as previously described [17]. In total, 3×10^4^ cells were seeded into each well of a 96-well Fluoplate (Nunc Rochester). At least six independent stable or transient transfections were made for each construct. Following the addition of luciferin to the culture medium, bioluminescence was assayed with a Topcount NXT counter (2-detector model, Perkin Elmer). Each well was counted for 3 s at intervals of ∼30 min. Plates were counted in an uninterrupted cycle. The counter was located in a thermostatically controlled room. During the experiments, cells were maintained in different lighting regimes. During the light phase, plates were illuminated between counting, with a tungsten light source (20 µW/cm^2^). Each trace represents the mean of at least two independent pools, each plated in a minimum of four wells. SD was also calculated and plotted. All assays were performed at least three times. Period and peak values were calculated as in previous studies [47],[48]. The original data were smoothed by an adjacent-averaging method with 4 h running means. The peak was calculated as the highest point of smoothed data, and the period was computed as the mean between the peaks in each cycle expressed as mean ± SD.

### qRT-PCR

Quantitative Real Time PCR analysis was performed using a StepOnePlus Real-Time PCR System (Applied Biosystems). Total RNA was reverse-transcribed into cDNA by using Superscript III Reverse Transcriptase (Invitrogen) with a mix of oligo dT and random primers. qRT-PCR conditions were 15 min at 95°C, then 40 cycles of 15 s at 95°C, 30 s at 60°C. The relative levels of *tef* mRNA were calculated by the 2-DDCT method. Relative expression levels were normalized to zebrafish *β-actin*.

### EMSA

TEF protein was obtained using the expression vector *pcDNA3.1-TEF* with the *TnT T7* Quick Coupled Transcription/Translation System from Promega according to the manufacturer's instructions. The in vitro translated TEF (0,5 µl) was pre-incubated for 10 min at room temperature in a 20 µl reaction containing 10% glycerol, 50 mM KCl, 10 mM HEPES (pH 7.6), 1 mM dithiothreitol, 0.1 mM EDTA, 5 mM MgCl_2_, 50 ng/µl poly[d(C-G)], and 50 mM Spermidine (Calbiochem). After pre-incubation, the DNA binding mixture was incubated for an additional 15 min with 25,000 cpm of the LRM DNA probe (from −187 to −137 of the zebrafish *per2* promoter) (5′-ggccatgggtgtccacgtgagttgtatgacacacttatgtaaaaagactgac-3′) labelled with γ^32^PATP using T4 Polynucleotide kinase.

The following competitor DNAs were added during the pre-incubation step, with a 50-fold molar excess with respect to the LRM probe.

LRM D-box (5′-gtatgacacacttatgtaaaaagactgac-3′), Sp1 (5′-attcgatcggggcggggcgagc-3′), LRM carrying a mutated D-box (5′-ggccatgggtgtccacgtgagttgtatgacacactcctcctagaagactgac-3′), and the LRM E-box (5′-ggccatgggtgtccacgtgagttgtatg-3′). The DNA binding mixtures were loaded and run for approximately 2 h on a 5% Polyacrylamide/Bis-acrylamide (37,5∶1) native gel in TEB 0.5× at 200 V after a pre-run at 50 volts for 30 min. The gel was then dried and exposed for autoradiography for at least 3 h.

## Supporting Information

Figure S1 **Co-localization of *per2*- and *aanat2*-driven expression in the pineal gland.** A double transgenic line was generated by crossing *Tg(-0.43per2:EGFP)tlv1* with *Tg(aanat2:mRFP)y164*, which exhibits red fluorescence specifically in the melatonin producing photoreceptor cells of the pineal gland. Confocal in vivo analysis reveals co-localized EGFP and mRFP expression in the pineal gland. mRFP (left panel), EGFP (right panel), and co-localized (middle panel) expression in the pineal gland are displayed in the figure.
(1.90 MB TIF)

Video S1 **EGFP expression in *Tg(-0.43per2:EGFP)tlv1*.** Confocal in vivo Z-stack of a 2-d-old *Tg(-0.43per2:EGFP)tlv1* embryos. The *per2* promoter drives ubiquitous EGFP expression that is augmented in the pineal gland. Thus *per2* promoter drives expression in virtually all peripheral clock-containing cells and expression is enhanced in the master clock located in the pineal gland.
(8.17 MB AVI)

## Abbreviations

CT: circadian time
DD: constant darkness
dpf: day(s) post-fertilization
EMSA: electrophoretic mobility shift assay
ISH: in situ hybridization
LD: light/dark
LL: constant light
LRM: light responsive module
MO: morpholino oligonucleotide
*per2*: *period2*
RHT: retino-hypothalamic tract
SCN: suprachiasmatic nucleus
TEF: thyrotroph embryonic factor
UTR: untranslated region
ZT: Zeitgeber time
