# Supplementary figures and images for: Light Directs Zebrafish period2 Expression via Conserved D and E Boxes
Source: PLoS Biol. 2009 Oct 27;7(10):e1000223. doi: 10.1371/journal.pbio.1000223 (PMC2759001; doi:10.1371/journal.pbio.1000223)

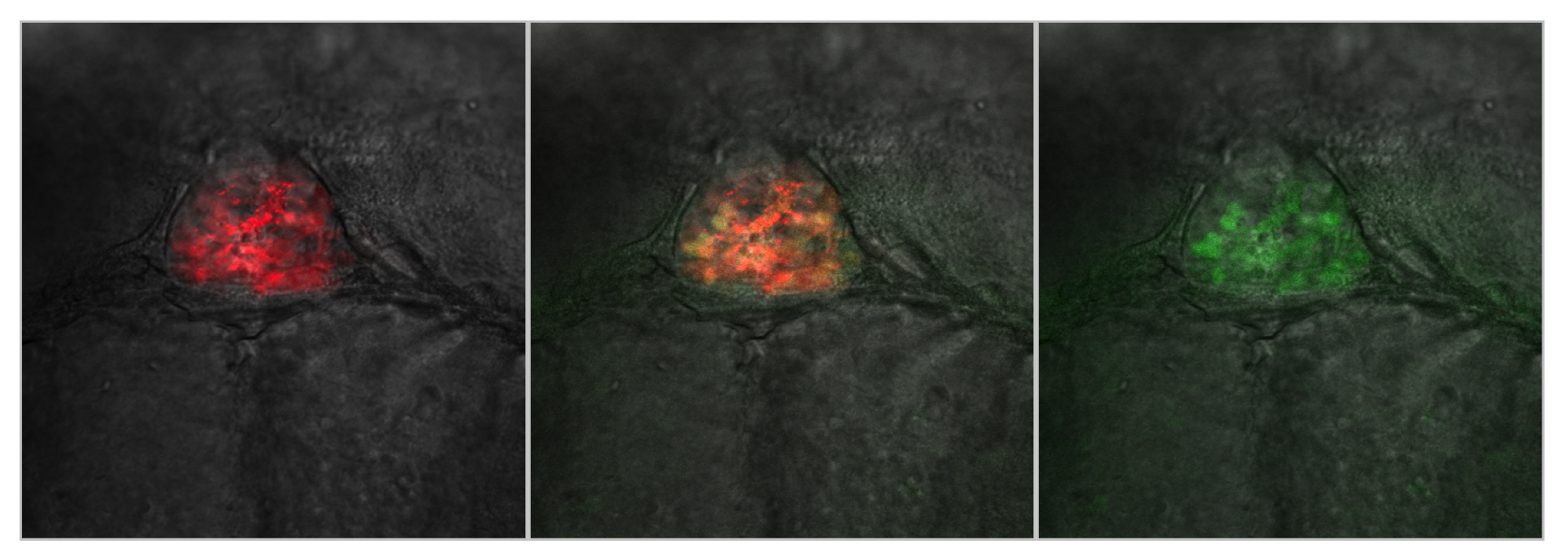

Supplement: Figure S1 — Co-localization of per2- and aanat2-driven expression in the pineal gland. A double transgenic line was generated by crossing Tg(-0.43per2:EGFP)tlv1 with Tg(aanat2:mRFP)y164, which exhibits red fluorescence specifically in the melatonin producing photoreceptor cells of the pineal gland. Confocal in vivo analysis reveals co-localized EGFP and mRFP expression in the pineal gland. mRFP (left panel), EGFP (right panel), and co-localized (middle panel) expression in the pineal gland are displayed in the figure. (1.90 MB TIF) [file pbio.1000223.s001.tif]
